# Desynchronisation of Glycolytic Oscillations in Yeast Cell Populations

**DOI:** 10.1371/journal.pone.0043276

**Published:** 2012-09-11

**Authors:** André Weber, Yury Prokazov, Werner Zuschratter, Marcus J. B. Hauser

**Affiliations:** 1 Biophysics Group, Institute of Experimental Physics, Otto von Guericke University, Magdeburg, Germany; 2 Special Laboratory for Electron and Laserscanning Microscopy, Leibniz Institute for Neurobiology, Magdeburg, Germany; Humboldt University, Germany

## Abstract

Glycolytic oscillations of intact yeast cells of the strain *Saccharomyces carlsbergensis* were investigated at both the levels of cell populations and of individual cells. Individual cells showed glycolytic oscillations even at very low cell densities (e.g. 1.0
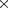
10^5^ cells/ml). By contrast, the collective behaviour on the population level was cell density-dependent: at high cell densities it is oscillatory, but below the threshold density of 1.0
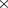
10^6^ cells/ml the collective dynamics becomes quiescent. We demonstrate that the transition in the collective dynamics is caused by the desynchronisation of the oscillations of individual cells. This is characteristic for a Kuramoto transition. Spatially resolved measurements at low cell densities revealed that even cells that adhere to their neighbours oscillated with their own, independent frequencies and phases.

## Introduction

Synchronisation is a wide-spread phenomenon of spontaneous self-organisation, where individuals coordinate their behaviour, such that collective, macroscopic dynamics emerges [1]–[3]. In synchronised populations of coupled oscillators, for instance cells, all individuals adhere to a collective, common rhythm. Typical examples of synchronisation in biological systems are the blinking rhythms of male fireflies [4], [5], the synchronisation of various circadian rhythms in mammals [6]–[8], the activity of brain areas [9], [10], the synchronisation of cells in the “segmentation clock” of vertebrates [11], the immune response to malaria parasites [12], [13], the onset of collective behaviour in social amoebae [14], and the macroscopic, collective glycolytic oscillations of yeast cells [15].

For unicellular organisms, such as yeast, life within a community is beneficial for the long-term survival [16]. Efficient cell-cell communication is a prerequisite for the organisation of communities, and such communication among individuals may be provided by metabolic oscillations.

Glycolysis is a fundamental pathway in the energy metabolism of eukaryotic cells and it may show oscillatory dynamics. Therefore, glycolytic oscillations of NADH and other metabolites that occur in populations of the yeasts *Saccharomyces carlsbergensis* and *Saccharomyces cerevisiae* have been intensively studied [17]–[21]. In dense populations, the individual cells synchronise their metabolism to a joint oscillatory mode [18], [22]. This has been demonstrated in experiments, where two sub-populations that oscillated with the same frequency, but at opposite phases, were blended and a new, collective oscillatory time-trace was recovered after a few minutes (i.e. after a few oscillatory cycles) [20], [23].

The coupling of individual cells is believed to be based on the relay and subsequent diffusion of messenger molecules through the extracellular medium, where they are absorbed by the other cells [23]. It is conjectured that mainly acetaldehyde plays the role of the coupling molecule [20]. In addition to studies in stirred cell suspensions, the propagation of waves of glycolytic activity has recently been observed in spatially extended media, such as settled cell suspensions [24] or gel-entrapped cells [25].

During the last two decades, substantial efforts have been made to develop realistic, detailed mechanistic models, which describe the glycolysis, its oscillations, and the cell-cell communication [26], [27]. Such models have been further used to study, for instance, the effect of intercellular coupling of yeast cells on the dynamics [26], [28]–[30], or the robustness of glycolytic oscillations towards internal and external fluctuations [30], [31].

Already early on, it has been observed that the dynamics of a cell population sensitively depends on cell density. At high cell densities yeast cells show synchronous, coherent oscillations. Once the cell density lies below a critical threshold, the yeast population no longer displays any collective oscillations, and rather remains quiescent [32].

The transition between collective oscillatory and stationary dynamics at the population level can follow two different mechanisms. The first of them, called ‘dynamical quorum sensing’ [33], [34], consists in the simultaneous cessation of oscillatory dynamics in every cell as the cell density drops below the critical threshold. Thus, the stationary dynamics at the collective level is identical to that of all cells of the population. Alternatively, the transition between quiescence and oscillatory dynamics at the collective, macroscopic level may be due to a so-called ‘Kuramoto transition’ [35], [36]. The collective oscillatory signal is generated at high cell densities, where the oscillations of all individual cells are coherent and synchronised, both in phase and oscillation period. At low densities, the yeast cells remain oscillatory, but they lose their coherence so that each of them oscillates with its own phase. As the collective behaviour is the sum of the single cell signals, phase-incoherent oscillations will lead to a stationary collective dynamics [37], [38].

How individual cells behave dynamically in low-density populations remains an important and still open question. While microscopy studies have indicated that the individual cells may still continue to oscillate at low cell densities [39], a recent study advocates that cells of *Saccharomyces cerevisiae* follow a ‘dynamical quorum sensing’ transition [33]. This interpretation is inferred from relaxation experiments, where, at low densities, cells are triggered to oscillate by an external stimulus, and the induced coherent oscillations were observed to decay in time [33]. Further support was drawn from studies in a flow-cell, where individual cells were quiescent and, again, could be stimulated by periodic addition of acetaldehyde to perform transient oscillations [40].

The present study aims to unravel the nature of the cell-density-dependent transition between oscillatory and stationary dynamics of intact yeast cells. The dynamics of immobilised cells of the yeast *S. carlsbergensis* is investigated at both the individual and collective level by using highly light- and position-sensitive single photon counting fluorescence detectors. We provide evidence that the dynamics in populations of *S. cerevisiae* is similar to that observed in *S. carlsbergensis*.

## Materials and Methods

Experiments were performed with aerobically grown yeast cells from *Saccharomyces carlsbergensis* (ATCC 9080; American Type Culture Collection, Manassas, VA, USA) cultivated aerobically in a rotary shaker (180 rpm) in liquid semisynthetic minimal medium [22] at 28°C. The cells were grown until the glucose in the medium was just exhausted at the transition from the logarithmic to the stationary growth phase. After harvesting the cells by centrifugation at 5000
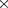
g at 21°C and washing them with distilled water, the wet cells were suspended in 0.1 M KH_2_PO_4_ buffer, pH 6.5, as a 20% (weight/volume) suspension and stirred at 23°C until they showed NADH oscillations. This generally occurred after 3–5 h of starvation. After starvation the cells were aliquoted in 1.5 ml Eppendorf cups and kept at 0°C for at most 3 days until use. Prior to measuring signals of cell populations, the aliquoted cells were diluted in 0.1 M KH_2_PO_4_ buffer, pH 6.5 to densities 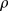 in the range of 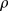 = 0.8–0.001% (w/v) (i.e. 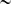
120
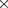
10^6^–0.1
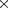
10^6^ cells/ml) and well aerated by stirring the suspension for at least 5 min.

Some experiments were conducted using cells of the yeast *Saccharomyces cerevisiae* diploid strain X2180. The cells were grown under aerobic conditions at 30°C in a rotary shaker (150 rpm) in a medium containing 10.0 g/l glucose, 6.7 g/l yeast nitrogen base (Bacto) and 100 mM potassium phthalate (Aldrich) at pH 5.0 until the glucose in the medium was exhausted. The yeast was harvested by centrifugation at 4066
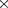
g for 3 min at 21°C, washed twice with 100 mM potassium phosphate buffer (Merck, Germany), pH 6.8 (centrifugation, 3 min at 4066
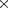
g) and re-suspended in the same buffer to a cell density of 10% (w/v). The cells were starved in suspension by shaking (30°C, 150 rpm) for 3 h and handled then as *S. carlsbergensis*, but with 100 mM potassium phosphate buffer (pH 6.8).

### Immobilisation of yeast cells in the batch chamber

Single yeast cells were immobilised on polylysine-coated coverslips. To this purpose, the coverslips were washed with acetone and distilled water. Subsequently, 100
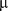
l of a 0.1 mg/l poly-D-lysine solution were spread on the coverslips, which were then dried in an oven at 50°C. Before use, they were carefully washed with water again and dried. Finally, a dry coverslip was fixed at the bottom of the batch chamber mounted above the objective of the inverted microscope.

The batch chamber consists of two parts between which the coated coverslip is clamped. 100
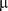
l well-aerated yeast suspension of a chosen cell density were placed into the chamber (consequently, the number of cells in the chamber varied according to the cell densities studied). After 30 min all cells settled and adhered to the coverslip when their plasma membranes got in contact with the polylysine coating. After sedimentation the experiment was started. To induce anaerobiosis 3 mM potassium cyanide were added, followed by an addition of 52 mM glucose 10 min later, which triggered the glycolysis and induced oscillations in the cells.

### Fluorescence microscopy

The intracellular dynamics was monitored through the autofluorescence of NADH which serves as an indicator for the glycolytic activity [22]. NADH is an intracellular metabolite which is directly involved in the glycolysis.

The NADH autofluorescence (absorption maximum at 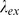 = 340 nm; emission maximum at 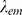 = 460 nm [42]) from single yeast cells was measured with an inverted Nikon Ti Eclipse (Nikon GmbH, Germany) microscope, equipped with a 100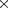/0.5–1.3 plan fluor lens and a position sensitive single photon counting photomultiplier tube as described previously [43]. For excitation of intracellular NADH a 8 MHz pulsed frequency-tripled Nd:vanadate laser tuned at 355 nm (HighQ Laser, Austria) was used. A dichroic mirror (z355rdc, AHF Analysentechnik, Germany) discriminated between excitation and emission. The emission light of yeast cells was filtered by a long-pass (LP 442 nm, Brightline, AHF, Germany) and a bandpass filter (FF01-440/40, Brightline, AHF, Germany) and detected by the photomultiplier. For all studied samples, the laser intensity was adjusted such that there was an incidence of 3,000–65,000 fluorescence photons/s at the detector, thus making sure that it operates under optimal conditions. The acquired photon positions were binned into frames of 512
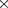
512 pixels, resulting in a resolution of 0.33
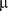
m/pixel in the object space. The field of view had a diameter of 169
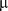
m. The photon flux was integrated over 2 s time intervals, which was a sufficient sampling rate to analyse glycolytic oscillations with periods in a range of 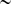
24–70 s.

### Image analysis

The global, collective signal of the whole population of yeast cells and the signal from individual cells were analysed separately. The immobilised yeast cells were randomly distributed on the coverslip. The collective signal was determined as the fluorescence light emitted from all cells in the entire area of observation. Therefore, all incident photons were summed up every second.

For the analysis of single cell signals, the position of each cell in the population was determined and its area, in pixels, marked. At any time, the single cell fluorescence 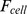 is the mean value of the intensity of the fluorescence signal detected in the area occupied by an individual cell
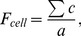
(1)where 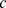 is the number of incident photons originating from the area 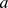 of the single cell. The temporal sequence of 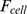 yields the time evolution of the fluorescence of an individual cell. For comparison, the amplitudes of the metabolic oscillations of the individual cells were normalised. Here, the norm was set by the oscillating amplitude of the cell in the population that showed the highest oscillatory amplitudes.

### Analysis of synchronicity within populations

From the time series of each cell in a population, we subtracted the baseline to eliminate spurious drifts and trends. The baseline was computed as the walking average of the fluorescence data using a time window corresponding to one period of oscillation. After baseline subtraction, we obtained oscillations around zero and determined their frequencies by a fast Fourier transform. The standard deviation 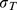 of the periods of oscillation of single cells was calculated as
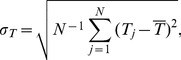
(2)where 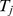 is the oscillation period of the 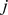
th cell, 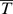 is the averaged period, and 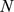 is the number of cells.

The noise in the time-series was filtered through a Fourier bandpass filter which cut off frequencies higher than 0.05 Hz and lower than 0.014 Hz. Thus, the frequencies of the glycolytic oscillations remained in the filtered time series 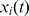. The phases 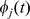 of each oscillating cell 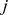


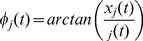
(3)were computed trough the Hilbert transform
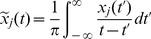
(4)of the filtered single cell signal 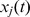. The order parameter K, introduced by Shinomoto and Kuramoto [38],
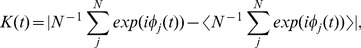
(5)and the time-averaged order parameter 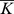


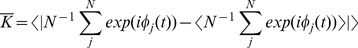
(6)were chosen for measuring the phase synchronisation. If the order parameter is close to 1 the coherence of oscillators in a population is high and if 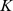 is 0 the cells oscillate at random phases. The standard deviation 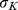 of 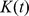 (eq. 5) and the mean value 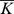 are used to quantify the phase synchronisation in each measurement.

## Results

The dynamics of intact cells of the yeast *S. carlsbergensis* has been studied for populations whose cell densities were varied from 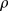 = 0.001% to 0.8%. The NADH fluorescence has been acquired over the entire field of view to monitor the collective, global behaviour of the yeast population. In addition, the dynamics of individual yeast cells in the population was also followed. Glycolytic oscillations in starved yeast cells were induced by addition of glucose aliquots to the cell medium. The oscillatory response of cells was long-lasting, however, always transient, since the experiments were performed under batch conditions.

**Figure 1:**
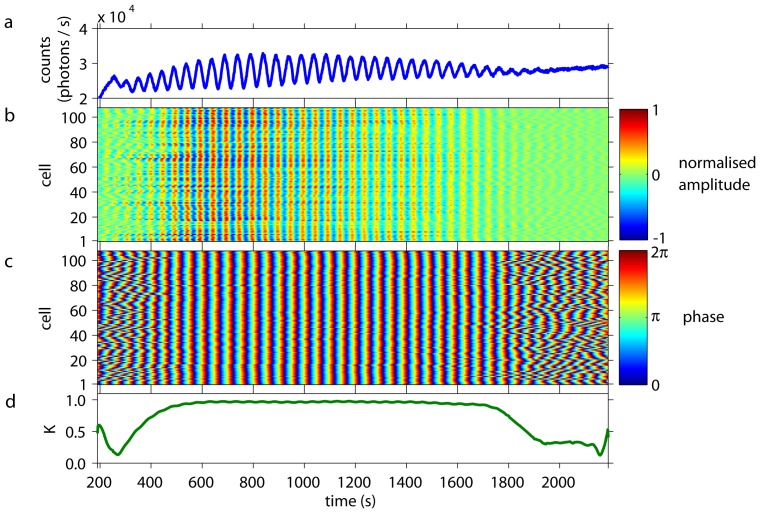
Collective and individual dynamics of a population of cells from *S. carlsbergensis* at a cell density of 0.7%. At 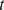 = 0 an aliquot of 52 mM glucose was added to the starved and immobilised cells. During the first 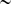
350 s of the time-series, the cells start to oscillate and to synchronise to each other. *(a)* The time-series of the macroscopic, collective fluorescence signal shows well-developed glycolytic oscillations. This coherent signal is generated by all cells of the population. *(b)* The oscillations of the individual cells show that their normalised amplitudes are highly coherent and synchronised. (The amplitude of oscillations of the cells corresponds to 60–140 photons/s.) Note that the dynamics of every cell is plotted as a thin line and its corresponding normalised amplitude is colour-coded. Cells are numbered randomly, i.e., the numbering does not reflect any spatial arrangement of the cells. At 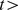
1800 s the oscillation amplitudes decay and the cells lose synchrony due to exhaustion of glucose. *(c)* The phases of the oscillations of the individual cells are highly synchronised to each other. Again, for 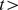
1800 s the cells desynchronise. *(d)* The time-dependent Kuramoto order parameter 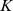 indicates the degree of synchronisation among the cells. For 350 s
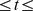
1800 s the order parameter is almost 1, indicating a very high coherence in the oscillations of the individual cells.

Dense cell populations showed collective glycolytic oscillations (Fig. 1a) of high amplitudes. Simultaneous analysis of individual cells revealed that their oscillations were synchronised in amplitude (Fig. 1b), frequency, and phase (Fig. 1c) to each other and to the collective oscillations (Video S1). The time-dependent Kuramoto order parameter 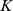 was close to 1, indicating a high degree of synchronisation and coherence of the cellular oscillations (Fig. 1d).

**Figure 2:**
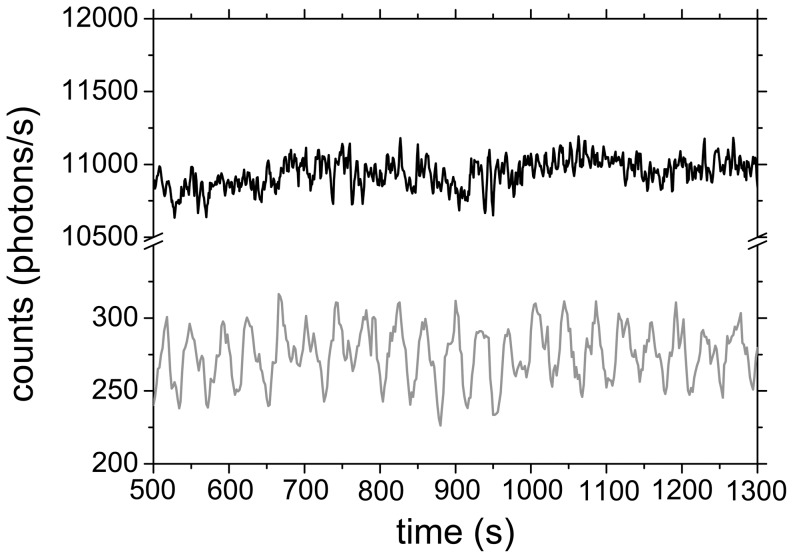
Dynamics of a *S. carlsbergensis* population of very low density ( 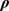
 **= 0.001%).** At 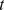 = 0 an aliquot of 52 mM glucose was added to the starved and immobilised cells. While the collective signal (black line) is quiescent and noisy, long-lasting oscillations are observed in the fluorescence signal emitted by an individual cell (gray line). The original time series were smoothed by adjacent averaging over three consecutive data points.

Collective, global oscillations can be observed down to cell densities of 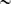
0.01%. Below this cell density, collective oscillations ceased, giving way to quiescent but often noisy time traces of NADH fluorescence (Fig. 2). However, the dynamics of individual yeast cells from low-density populations (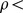
0.01%) remained oscillatory (Video S2). In fact, these individual oscillations were clearly detectable and long-lasting, even in very sparse populations (Fig. 2, where 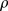 = 0.001%).

**Figure 3:**
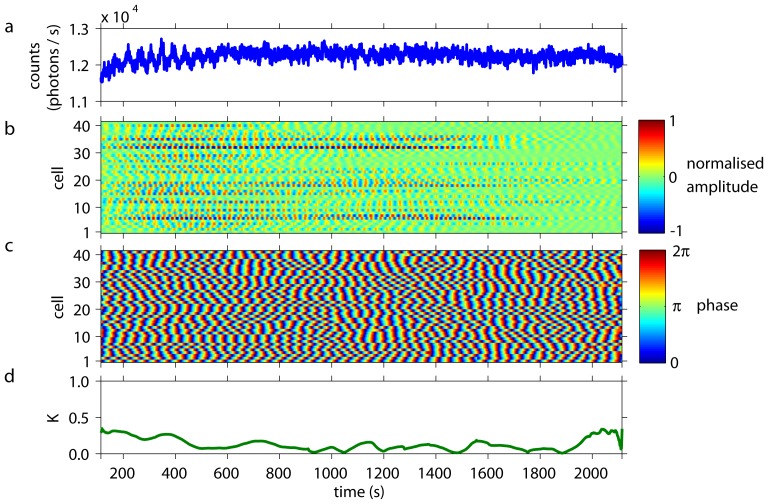
Collective and individual dynamics of *S. carlsbergensis* at a cell density of 0.01%. At 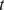 = 0 an aliquot of 52 mM glucose was added to the starved and immobilised cells. *(a)* The time-series of the macroscopic, collective fluorescence signal is noisy but quiescent. Transient reminicences of oscillations can be spotted at 200 s
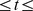
400 s. *(b)* The individual cells remain oscillatory, even at this low cell density. The normalised oscillation amplitudes of the individual cells are decoherent. The amplitude of the oscillations of the cells corresponds to 120–200 photons/s.) Note, that the time spans where individual cells show pronounced oscillatory amplitudes vary considerably from cell to cell. The cells are numbered in a random order. *(c)* The plot of the phases of the oscillations shows that each cell oscillates, however, with its own phase and oscillation period. A slight and transient entrainment in the oscillation phases may be observed for some cells, giving rise to the reminicences of oscillations seen at collective signal at 200 s
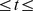
400 s. *(d)* The time-dependent Kuramoto order parameter 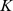 remains low at all times, indicating a complete desynchronisation among the oscillations of the individual cells.

The typical behaviour of yeast cells at very low cell densities is presented in Fig. 3 and Video S2, where 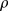 = 0.01%. Each cell oscillated with its own phase (Fig. 3c) and the phases of the individual cells did not synchronise. Furthermore, the amplitudes of the metabolic oscillations varied, and so did the instants, when the oscillations set in in each individual cell (Fig. 3b). The low values of the order parameter 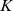 (Fig. 3d) indicated decoherence between the dynamics of the oscillating cells. Consequently, the collective fluorescence signal of the entire population showed stationary behaviour (Fig. 3a).

**Figure 4:**
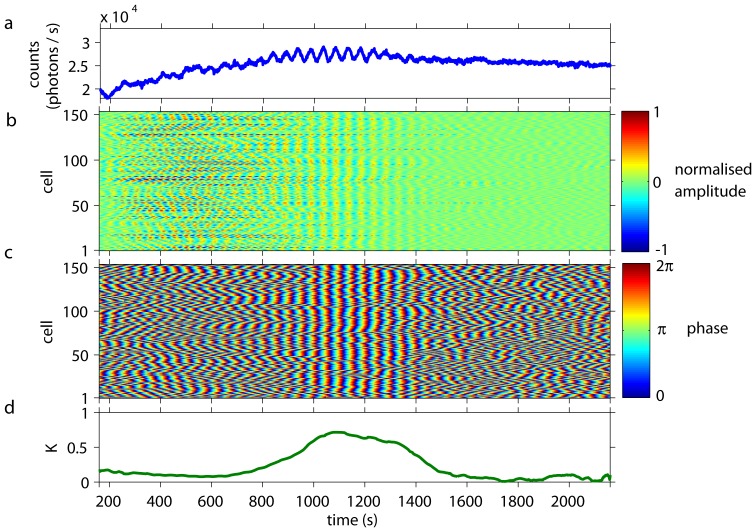
Collective and individual dynamics of *S. carlsbergensis* at a cell density of 0.1%. At 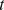 = 0 an aliquot of 52 mM glucose was added to the starved and immobilised cells. *(a)* The time-series of the macroscopic, collective fluorescence signal is quiescent. At 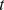 = 850 s a transient episode of synchronised, collective oscillations sets in. After 1350 s, the collective signal becomes quiescent again. *(b)* The individual cells remain oscillatory during the entire duration of the experiment. Remarkably, the synchronisation episode sets in as the normalised amplitudes of the glycolytic oscillations already diminishes. (The amplitude of the oscillations of the cells corresponds to 45–95 photons/s.) The cells are numbered in an random order. *(c)* At the begin and end of the experiment, the individual cells oscillate at their own periods and phases. The synchronisation episode at 850 s
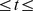
1350 s is caused by a temporary entrainment of the oscillations of the individual cells. *(d)* The time-dependent Kuramoto order parameter reflects the dynamics in the cell population. It remains below 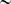
0.3 as the cells oscillate in a incoherent manner, and increases to levels of 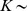
0.7 during the synchronisation episode.

Experiments were performed at different cell densities to find the transition between synchronised metabolic oscillations and the loss of synchrony as function of the population density. As the cell densities were decreased to 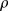 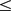
0.3%, the coherence among the cells was found to diminish. Below 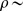
0.01% collective oscillations ceased, whereas the individual cells remained oscillatory. Fig. 4 illustrates the situation at an intermediate cell density, 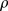 = 0.1%. Here, the cells began to oscillate with large amplitudes immediately after glucose addition (Fig. 4b), however, there was no defined phase relation among these oscillations (Fig. 4c) for the first 850 s after delivery of glucose. Consequently, the global signal is quiescent (Fig. 4a). After 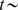
850 s (Fig. 4), these oscillations synchronised their phases and yielded some collective oscillations (850 s
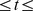
1350 s), before phase desynchronisation set in again. An inspection of the oscillations of the individual cells revealed that their amplitudes were already decaying as the episode of phase synchronisation began (Fig. 4b). The order parameter 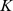 follows the trends outlined above, and it transiently reached higher levels during the episode of phase synchronisation (Fig. 4d).

The experiments show that while the individual cells remained oscillatory at all cell densities studied (i.e., 0.001%
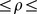
0.8%), the collective behaviour changed depending on the cell density. For very dense populations, i.e., 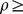
0.3%, the cellular oscillations were fully synchronised, leading to well developed collective oscillations. At intermediate densities (0.01%
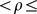
0.3%) the cells became increasingly decoherent as 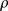 decreased, yielding transient episodes of collective oscillations. Finally, at very sparse populations (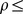
0.01%) the collective dynamics was always quiescent.

**Figure 5:**
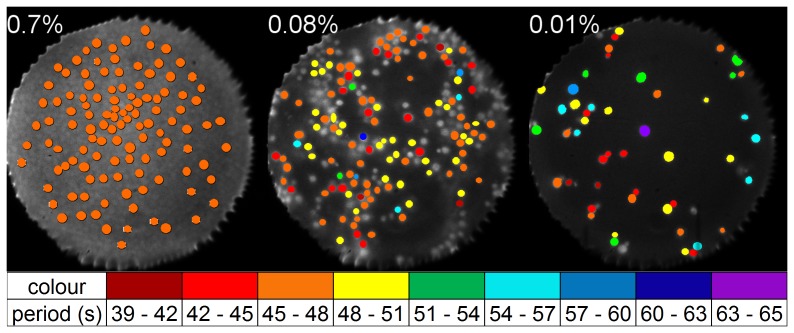
Mapping of the oscillation periods of individual cells in a population, for three experiments at cell densities of 0.7%, 0.08% and 0.01%, respectively. While for high cell densities (e.g. 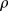 = 0.7%; not all cells labelled) all cells show same the oscillation period, the cells lose coherence in oscillation periods at lower cell densities (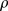 = 0.08% and 0.01%). Regions, where all cells of the cluster oscillate with same period, are not found in populations of low densities (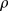 = 0.08% and 0.01%). At densities below 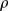 = 0.3%, domains devoid of cells are present in the areas of observation. Marked cells without colouration did not show distinctive oscillations. The field of view has a diameter of 169
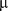
m.

To investigate whether spatial effects played a role during the loss of synchrony, the oscillation frequencies of individual cells were determined. An overview of oscillation periods of single cells and their spatial distributions is shown in Fig. 5. In dense populations, individual cells displayed oscillations that lay within a narrow frequency band, whereas the frequency band broadened as 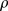 decreased below 0.3%. In such populations, we did not find any domains, where all cells oscillated with the same frequency (or period). Instead, in some cases we observed that even adjacent cells oscillated with different periods (Fig. 5b,c).

**Figure 6:**
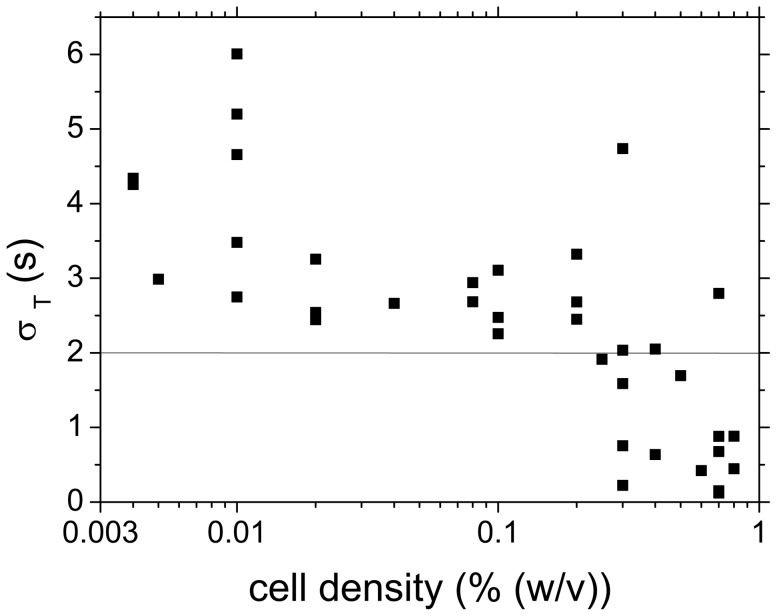
Width of the distribution of oscillation periods in individual experiments, as measured by the standard deviation 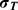
 **of the Gaussian fit to the histogram of oscillation periods.** For cell densities 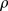 below 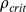 = 0.3%, 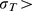
2 s pointing at decoherence in oscillation periods. The line at 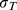 indicates the sampling rate (2 s). All points below 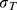 = 2 s originate from coherent oscillations of the individual cells.

The loss of frequency coherence among the cells in low-density populations (Fig. 5) led to a spread of oscillation periods. Their distribution was found to be Gaussian and the standard deviation 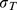 was used as a measure for the coherence of the glycolytic oscillations. The standard deviation 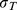 of the oscillation periods in dependence of the cell density is compiled in Fig. 6. While at 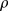 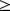
0.3% the distributions of oscillation periods remained narrow (i.e., 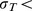
2 s), the distributions broadened, as 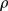 fell below 0.3%. As the sampling rate of the analysis was 2 s, any value of 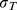 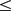
2 s indicates synchronisation of the oscillations of individual cells in the population.

**Figure 7:**
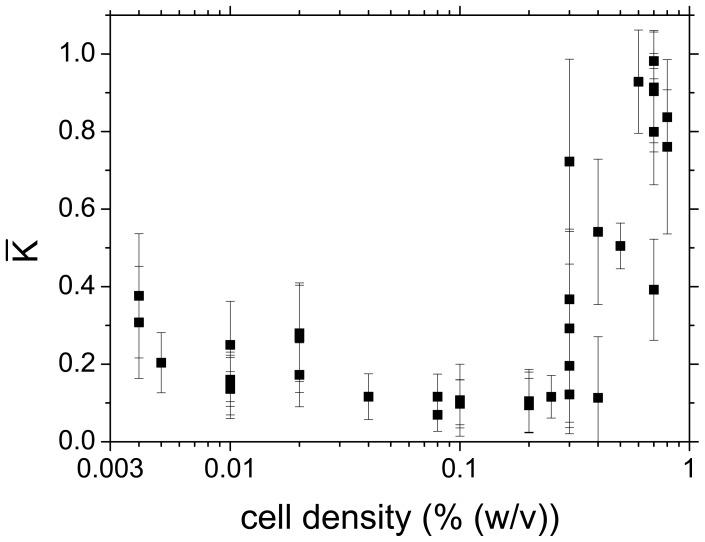
Time-averaged Kuramoto order parameter 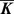
 **for experiments at different cell densities** 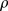 **.** At cell densities above 0.3%, 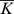 is high indicating coherent oscillatory dynamics of the cells in the population. At cell densities below 0.3% the low value of 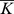 shows that the cells oscillate in a decoherent fashion. The critical cell density lies at 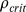 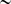
0.3%. The error bars represent the standard deviation 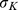 and provide a measure for the fluctuations of the phase synchronisation in each individual experiment.

Alternatively, the transition from synchronised to desynchronised behaviour was determined by calculating the time-averaged order parameter 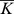 for each experiment (Fig. 7). Again, the phase synchronisation for 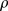 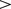
0.3% was high (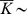
1), whereas a decrease of 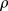 below 0.3% led to a loss of coherence, which is reflected by the small values of 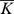. Very close to the critical cell density 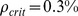, the standard deviations 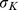 of the Kuramoto parameter 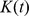 increased, since the oscillation phases of the individual cells desynchronised transiently.

## Discussion

**Figure 8:**
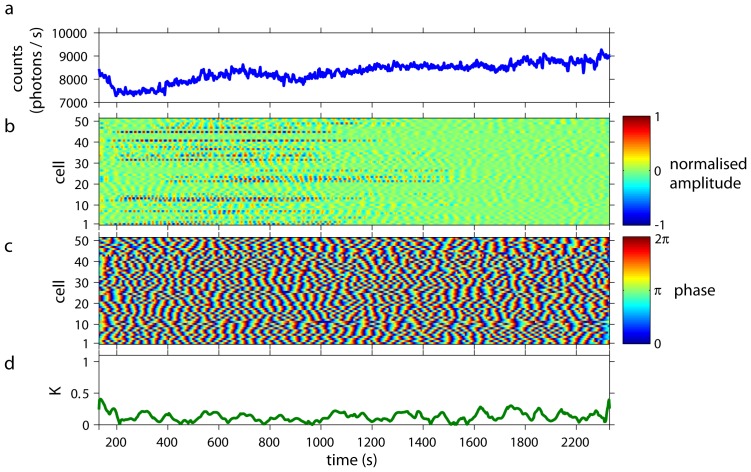
Collective and individual dynamics of *S. cerevisiae* at a cell density of 0.04%. At 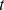 = 0 an aliquot of 52 mM glucose was added to the starved and immobilised cells. *(a)* The time-series of the macroscopic, collective fluorescence signal is quiescent. *(b)* The individual *S. cerevisiae* cells remain oscillatory, even at this low cell density. The normalised oscillation amplitudes of the individual cells are decoherent. The amplitude of the oscillations of the cells corresponds to 15–45 photons/s.) Note, that the duration and the onset of pronounced oscillation amplitudes in the individual cells vary considerably from cell to cell. The cells are numbered randomly. *(c)* The plot of the phases of oscillations shows that each cell oscillates with its own phase and oscillation period. *(d)* The time-dependent Kuramoto order parameter 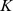 remains low, indicating a complete desynchronisation among the oscillations of the individual cells.

More than three decades ago, the question has been risen, what happens on the single cell level, when the macroscopic, collective oscillations in yeast cell populations disappear [32]. For immobilised cells of the yeast *Saccharomyces carlsbergensis*, we now provide an answer: The individual cells continue to oscillate, even if the collective oscillations cease and the macroscopic dynamics becomes quiescent. The oscillatory dynamics of individual cells persists for very low cell densities (down to 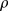 = 0.001%), where the cells are almost isolated. To determine whether the persistence of oscillations at extremely low cell densities is a specific feature of *S. carlsbergensis* or whether this behaviour is a general property of yeast cells, we repeated the studies using another yeast strain, namely *S. cerevisiae*. Again, the oscillations at the level of individual cells were maintained, even at such low cell densities, where the collective dynamics was stationary. As in *S. carlsbergensis*, the glycolytic oscillations of individual cells of *S. cerevisiae* in low-density populations are desynchronised from each other (Fig. 8). Thus, we conclude that the persistence of oscillations at the cellular level at such low cell densities, where the collective behaviour is quiescent, is a general dynamic feature of yeasts.

The disappearance of collective glycolytic oscillations at low cell densities is due to a loss of dynamic coherence among the individual cells. Below the critical density 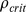 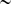
0.3%, the cells continue to display glycolytic oscillations, but these oscillations lose their synchrony in phase and frequency. This desynchronisation scenario is typical for a Kuramoto transition to incoherence (or desynchronisation) [35], [37], [38]. It differs significantly from the recently expressed view that the cessation of collective oscillations at the population level is due to a ‘dynamical quorum sensing’ phenomenon [33], where all cells stop oscillating immediately, once the cell density is lowered below the critical threshold.

The cessation of collective oscillations occurs due to the inability of the cells to synchronise, since the extracellular messenger molecule acetaldehyde no longer reaches the necessary extracellular concentration to ensure an effective cell-to-cell coupling (communication). Interestingly, the critical cell density, where the transition in collective dynamics occurs, coincides with the emergence of small spots devoid of cells in the probe (Fig. 5). This coincidence may rise the question, whether the cell-to-cell communication between yeast cells is indeed achieved via the extracellular (mean-field) concentration of the messenger molecule (acetaldehyde), or whether a direct contact between the cells is necessary for the synchronisation to take place.

An indication of which mechanism is effective can be obtained from experiments at low cell density, e.g. 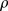 = 0.01%. The cells were sparsely distributed on the coverslip, most of them isolated from each other, while another couple of cells stuck together via membrane-membrane contact (see the field of view for 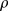 = 0.01%, Fig. 5c). It is noteworthy, that each of the adherent cells showed oscillations which differed in oscillation period and phase from that of the cell adhering to it (Fig. 5c: the three pairs of adhering cells in the centre of figure). The absence of a common frequency of oscillations in adhering cells provides support for the concept that yeast cells may indeed be coupled via the extracellular concentration of a messenger molecule.

On the other hand, at very low cell densities, a small fraction of cells remained quiescent. The reasons for this behaviour are not yet known, however, it might be due either to the intrinsic dynamics of these cells, or due to a “non-local” coupling in such reaction-diffusion systems, which suppresses their oscillations [41]. Whether such a mechanism is effective in sparse yeast cell populations will be investigated in future.

Two measures have been introduced to quantify the degree of synchronisation among the glycolytic oscillations at the cellular level. While 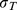 quantifies the synchronisation of the frequencies of the individual cells, 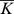 measures the coherence of their oscillation phases. Both measures, 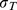 as a measure for the widths of the distributions of oscillatory periods (Fig. 6) and the time-averaged order parameter 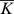 (Fig. 7), independently show that the transition between synchrony and desynchronisation of the individual cells of a yeast population takes place at a cell density 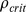 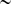
0.3%.

The value of the critical cell density 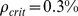 lies much higher than the cell density below which the collective dynamics settles onto a quiescent behaviour (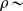
0.01%). This apparent discrepancy is due to the fact that 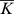 and 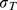 very accurately define the onset of desynchronisation (at the phase transition between synchronisation and desynchronisation), while the transition to collective quiescent behaviour is due to a complete decoherence of the oscillations of the individual cells. In future, we wish to analyse the transition from decoherence to the onset of coherence by making use of recently introduced techniques of synchronisation analysis [9].

The Kuramoto parameter 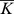 and its standard deviation 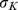 tend to slightly increase as the cell density approaches zero (Fig. 7), although the cells continued to show glycolytic NADH oscillations. This finding reflects the loss of accuracy of the statistical measure 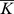 as the number of cells becomes very low, i.e., as the cell density 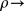
0. The increases in 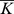 and 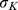 reflect the occurrence of intermittent episodes where a pair of cells may transiently oscillate with a comparable frequency.

## Conclusions

The present study provides an answer for the long-standing, open question, how individual yeast cells behave, when the cell density falls below a critical value and the macroscopic, collective oscillations of the population cease [32]. We found that, while the collective dynamics of the entire population changes from oscillatory to stationary, the individual cells in these populations remained oscillatory. Such dynamics was even observed at the very low cell density of 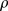 = 0.001%, where the cells are isolated from each other. However, at very diluted cell populations the oscillations of the individual cells became desynchronised. This behaviour was observed in populations of both yeasts *S. carlsbergensis* and *S. cerevisiae*, indicating that the described dynamic transition is a generic feature of yeast.

The loss of coherence in the global, collective dynamics occurs via a ‘Kuramoto transition’, where the oscillations of the individual cells lose their phase and frequency coherence. In populations of low densities even cells adhering to each other showed oscillations that differed in frequency and period, thus, no spatial clusters, where all cells oscillated with the same frequency, were observed. These new findings need to be taken into account in new modelling studies of the transition between synchronisation and desynchronisation of yeast cell populations, especially since more recent studies in stirred suspensions tended to favour the ‘dynamical quorum sensing’ mechanism, where all cells stop to oscillate as the global, collective oscillations vanish.

## Supporting Information

Video S1
**Synchronised oscillatory dynamics of a population of cells from** ***S. carlsbergenis*** **at a cell density of 0.7%.** The oscillatory behaviour of a dense cell population (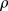 = 0.7%) in a 3D representation. The 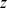-axis codes for the fluorescence intensity which is detected at any 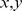-position of the sample. The fluorescence intensity emitted by all cells oscillates, and these oscillations are well synchronised in phase and frequency.(GIF)

Video S2
**Desynchronised oscillatory dynamics of a population of cells from** ***S. carlsbergenis*** **at a cell density of 0.01%.** The oscillatory behaviour of a sparse cell population (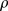 = 0.01%) in a 3D representation. The 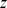-axis codes for the fluorescence intensity which is detected at any 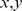-position of the sample. It can be seen that, while all individual cells show metabolic NADH oscillations, these oscillations are desynchronised in phase and frequency. Thus, each cell oscillates at its own pace.(GIF)
