# Supplementary figures and images for: Desynchronisation of Glycolytic Oscillations in Yeast Cell Populations
Source: PLoS One. 2012 Sep 11;7(9):e43276. doi: 10.1371/journal.pone.0043276 (PMC3439430; doi:10.1371/journal.pone.0043276)

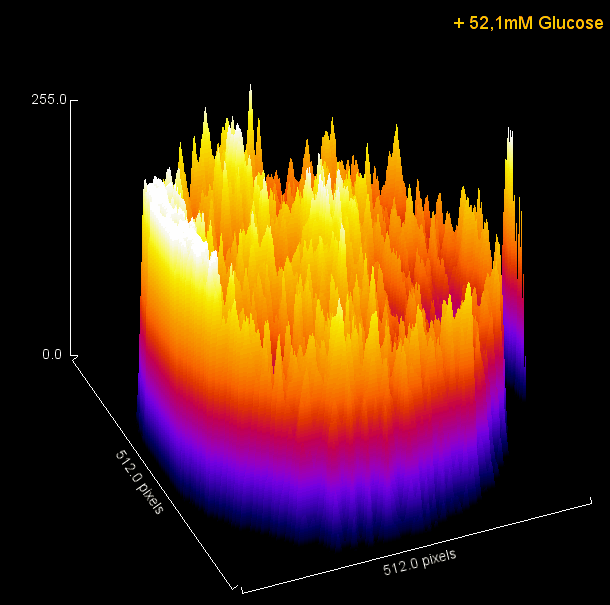

Supplement: Video S1 — Synchronised oscillatory dynamics of a population of cells from S. carlsbergenis at a cell density of 0.7%. The oscillatory behaviour of a dense cell population ( = 0.7%) in a 3D representation. The -axis codes for the fluorescence intensity which is detected at any -position of the sample. The fluorescence intensity emitted by all cells oscillates, and these oscillations are well synchronised in phase and frequency. (GIF) [file pone.0043276.s001.gif]

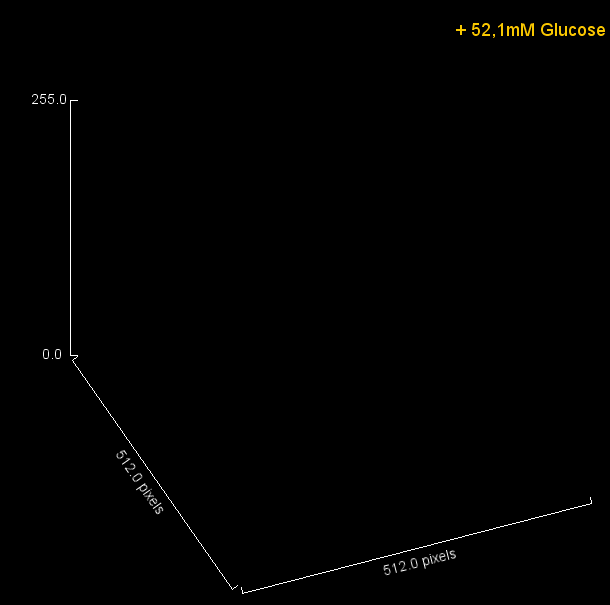

Supplement: Video S2 — Desynchronised oscillatory dynamics of a population of cells from S. carlsbergenis at a cell density of 0.01%. The oscillatory behaviour of a sparse cell population ( = 0.01%) in a 3D representation. The -axis codes for the fluorescence intensity which is detected at any -position of the sample. It can be seen that, while all individual cells show metabolic NADH oscillations, these oscillations are desynchronised in phase and frequency. Thus, each cell oscillates at its own pace. (GIF) [file pone.0043276.s002.gif]
